# Impaired migratory phenotype of CD4^+^ T cells in Parkinson’s disease

**DOI:** 10.1038/s41531-022-00438-0

**Published:** 2022-12-10

**Authors:** Dejan Mamula, Shervin Khosousi, Yachao He, Vesna Lazarevic, Per Svenningsson

**Affiliations:** 1 Department of Clinical Neuroscience, Karolinska Institutet, Stockholm, Sweden; 2 Basic and Clinical Neuroscience, King’s College London, London, United Kingdom

**Keywords:** Neuroimmunology, Neuroimmunology

## Abstract

Dysfunctions in the immune system appear implicated in both disease onset and progression of Parkinson’s disease (PD). Neurodegeneration observed in the brain of PD patients has been associated with neuroinflammation that is linked to alterations in peripheral adaptive immunity, where CD4^+^ T cells are key players. In the present study, we elucidated the immunological aspect of PD by employing a wide range of cellular assays, immunocytochemistry and flow cytometry to examine CD4^+^ T cells. We particularly investigated the role of CD4^+^ T cell migration in the proper functioning of the adaptive immune system. Our data reveal the altered migration potential of CD4^+^ T cells derived from PD patients, along with impaired mitochondrial positioning within the cell and reduced mitochondrial functionality. In addition, a cross-sectional study of p11 levels in CD4^+^ T cell subsets showed a differentially increased level of p11 in Th1, Th2 and Th17 populations. Taken together, these results demonstrate major impairments in the functionality of peripheral CD4^+^ T cells in PD.

## Introduction

Parkinson’s disease (PD) is the second most common neurodegenerative disorder, with up to 1% of prevalence among people older than 60^1^. PD is clinically diagnosed based on the presence of bradykinesia, rigidity and tremor. PD patients also suffer from a multitude of non-motor symptoms, including sleep disorders, depression and cognitive impairments, largely contributing to poor quality of life^2^. The major neuropathological hallmarks of PD are the loss of dopaminergic neurons in substantia nigra and the formation of neuropathological inclusions, named Lewy bodies and Lewy neurites^3^. The therapeutic approaches towards PD are symptomatic replacement of dopaminergic neurotransmission, but they do not slow down PD progression^4^.

Important progress towards understanding etiology and progression of PD, and other neurodegenerative disorders, has been made by widening the focus of research to the critical involvement of the immune system^5^. For example, PD patients have been shown to have increased microgliosis, elevated levels of peripheral proinflammatory cytokines, higher risk for auto-immune diseases, and aberrant adaptive immune responses^6^. Also, it has been shown that particular major histocompatibility complex (MHC) alleles linked with PD, display α- synuclein derived peptide epitopes, thereby inducing T cell-mediated immunity in PD patients^7^. The adaptive immune system includes different cell types with the distinctive role of CD4^+^ T cells which is usually observed through the interaction with other immune cells in order to cope with environmental challenges^8–10^. PD-associated transcriptomic signatures, for example, oxidative stress and mitophagy, was found in CD4^+^ memory T cells suggesting their association with disease pathogenesis^11^.

During random migration, CD4^+^ T cells need to optimally scan their environment to properly exert their function and this process is critically influenced by velocity and directionality^12^. The velocity and directionality of CD4^+^ T cells are regulated by specific molecular signaling to GTPases and cofilin^13^. Lymphocyte migration is highly dependent on the proper positioning of mitochondria at the uropod with their localized ATP production^14–16^. Mitochondria are indispensable for facilitating chemotactic lymphocyte movement and mitochondrial reactive oxygen species (mROS) play an important role in proper T cell activation and proliferation^17^. It has been shown that mROS amplifies T- cell receptor (TCR) proximal signaling and that transient formation of mROS is important for the activation of certain transcription factors^18–21^. In the case of mitochondrial DNA deficit, mROS are reduced, subsequently leading to a decrease in the expression of the cytokines IL-2 and IL-4^20^. It has been shown that ROS promotes polarization of anti-CD3/CD28 primed human T cells into Th2 phenotype^22^. Interestingly, PD patients demonstrate Th1 bias^23^. However, the involvement of ROS and underlying cellular mechanisms of CD4^+^ T cells in disease progression are poorly understood.

A long latency between the first pathological changes and clinical diagnosis of PD requires finding reliable molecular and cellular biomarkers. We previously showed that p11 has an elevated expression in distinctive leukocyte populations in PD patients^24^. p11 (S100A10, annexin II light chain) together with Annexin A2 constitute a heterotetrameric protein complex localized preferentially at cell membranes which coordinates multiple cellular processes^25^. Annexin A2 regulates actin bundling and couples cytoskeleton with plasma membrane^26^. The neuronal function of p11 has been implicated in PD and L-dopa responses^27^ but also in the pathophysiology of depression and antidepressant therapies^28^. Increased levels of p11 in specific subsets of peripheral leukocytes isolated from PD patients, introduced p11 as a potential biomarker, primarily in PD patients with associated depression^24^.

The aim of the present study was to further elucidate immunological aspects of PD, focusing primarily on CD4^+^ T cells. To this end, we evaluated the migratory phenotype of PD-derived CD4^+^ T cells and underlying cellular events. Additionally, we carried out a cross-sectional study of p11 expression in different CD4^+^ Th subsets in attempt to come closer to the application of p11 as a potential inflammation-related PD biomarker.

## Results

### Subjects

The study included 30 healthy controls and 49 PD patients from neurological clinics in Region Stockholm (Table 1 and Supplementary Tables 1, 2). All research participants gave their consents according to the Declaration of Helsinki further approved by the regional ethical committees. Clinical diagnostic criteria for PD^29^ were fulfilled by all participants. PD severity was scored using both Unified Parkinson’s Disease Rating Scale (UPDRS) and Hoehn and Yahr (H&Y) scale. Montreal Cognitive Assessment (MoCA) scores was used for cognitive assessment, Montgomery Åsberg Depression Rating Scale (MADRS) was used to assess depression, and levodopa equivalent daily dose (LEDD) for a total amount of dopaminergic medication^30^.

**Table 1:** Demographical and clinical characteristics of all subjects who participated in this research.

PBMCs								
Group	Sex (M/F) (%)	Age mean ± SD	Disease duration (years) median (range)	H&Y score median (range)	MDS- UPDRS III median (IQR)	MoCA median (IQR)	MADRS median (IQR)	LEDD median (IQR)
Control (*n* = 30)	43/57	64,4 ± 9,5	n/a	n/a	n/a	n/a	n/a	n/a
PD (*n* = 49)	59/41	67,3 ± 10,2	4,3 (0,0–21,1)	2 (1–5)	36 (25,5–43,5)	26 (24,5–28)	8 (4–13)	610 (160–937,5)

Measures are presented as mean ± standard deviation, median (range), or median (interquartile range [IQR]).

*PD* Parkinson’s disease, *F* female, *M* male, *n/a* not applicable, *H&Y* Hoehn and Yahr scale, *UPDRS* unified Parkinson’s disease rating scale, *MoCA* Montreal cognitive assessment, *MADRS* Montgomery-Åsberg depression rating scale, *LEDD* L-dopa equivalent daily dose, *PBMCs* peripheral blood mononuclear cells.

Control subjects were selected among healthy volunteers and were age- and sex-matched to PD patients (Table 1). Subjects with systemic inflammatory disorders or anti-inflammatory therapies were excluded.

### Migratory deficit of PD-derived CD4 + T cells

Several lines of evidence propose that immune system dysregulations, not least in Th cells, play an important role in PD pathogenesis^31,32^. Taking into account that efficient immune response highly depends on the directional migration of lymphocytes, we focused our study on the assessment of the migration potential of CD4^+^ T cells. Hence, we employed a transwell assay to measure the ability of CD3/CD28-activated CD4^+^ T cells to migrate in in vitro conditions. For this experiment, cells from 11 PD and 11 age and sex-matched controls were used.

**Fig. 1:**
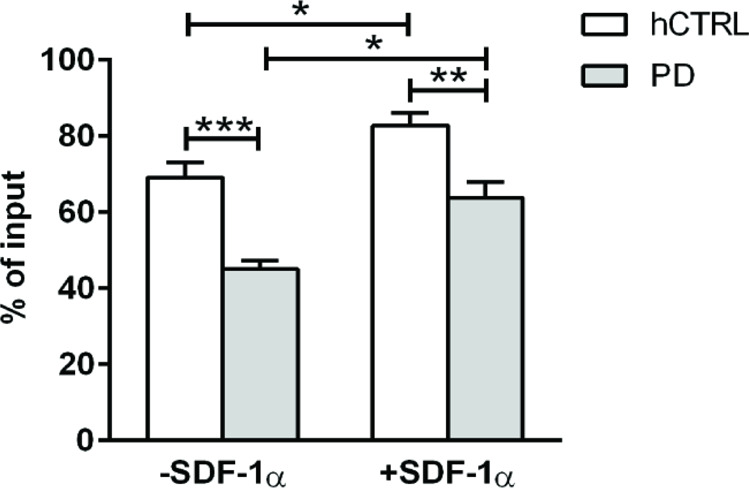
Decreased motility of PD patients derived primary CD4^+^ T cells. Transwell migration of primary CD4^+^ T cells derived from 11 PD patients and 11 age/sex-matched healthy controls with or without SDF-1α. Transmigration to the lower chamber was assayed using ICAM-1 coated transwells. Cultured CD4^+^ T cells were allowed to migrate for 1 h at 37 °C to the lower chamber and the number of migrated cells was counted by FACS. The data were obtained from four independent experiments. The percentages of migrated cells to input cells for each group and condition are expressed as the mean percentage ± SEM. Statistical significance was assessed by using two-way ANOVA followed by Tukey’s multiple comparisons test. *p < 0.05, **p < 0.01, ***p < 0.001.

As shown in Fig. 1, both PD and healthy control (hCTRL) derived activated CD4 + T cells readily migrated to the lower chamber with and without the addition of a chemoattractant (stromal cell-derived factor-1α, SDF-1α). However, the percentage of input of PD-derived CD4^+^ T cells that migrated to the lower chamber was significantly lower compared to hCTRL in both conditions suggesting a migratory deficit in these cells (Interaction, *F*(1, 52) = 0.4887 *p* = 0.4876; SDF-1α, *F* = 19, 32 *p* < 0.0001; PD, *F* = 34, 13 *p* < 0.0001).

**Fig. 2:**
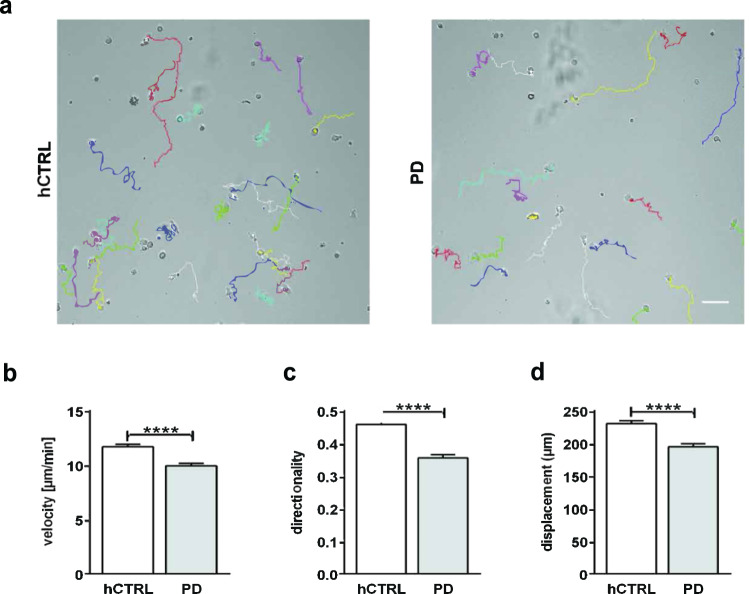
Primary CD4^+^ T cells derived from PD patients are slower and less persistent than healthy controls. Primary CD4^+^ T cells derived from nine PD patients and nine age/sex-matched healthy subjects were cultured for 7 days. The cells were transferred to 10 μg/mL human ICAM-1 coated µ-slides and incubated at 37 °C, 5% CO_2_ for 45 min. Live imaging was performed while cells were migrating on ICAM-1 coated 2D surface. Both PD and control CD4^+^ T cells were recorded at 10x magnification for 20 min and subsequently manually tracked. **a** Representative snapshots with cell tracks for both PD and control (arbitrary colors); scale bar, 50 μm. **b**–**d** Quantification of velocity (**b**), directionality (**c**), and displacement (**d**) of all tracked cells. The data were obtained from nine independent experiments (345 PD cells and 293 healthy control cells were tracked). Represented data are means ± SEM. Significance was assessed by Mann–Whitney test. ****p < 0.0001.

To further explore the observed phenotype we proceeded with a 2D migration assay on ICAM1 substrate (Fig. 2a). This allowed us to characterize not only migration potential of cells but also migratory parameters such as velocity and directionality. As shown in Fig. 2, our data revealed both reduced velocity (hCTRL 11.69 ± 0.23 µm/min vs PD 9.94 ± 0,22 µm/min, *p* < 0,0001; Fig. 2b) and directionality (hCTRL 0.458 ± 0.012 vs PD 0.355 ± 0.010 *p* < 0,0001; Fig. 2c) of PD-derived CD4^+^ T cells. Consequently, that also led to a reduction of displacement (hCTRL 230.1 ± 4.54 µm vs PD 194.9 ± 4.36 µm, *p* < 0.0001; Fig. 2d), confirming altered migratory properties of PD-derived CD4^+^ T cells.

We finally investigated whether the velocity and directionality of the PD-derived CD4 + cells were affected by disease stage, disease duration or levodopa medication, but found no significant correlations (Supplementary Fig. 1, Spearman’s rho *p* > 0.05).

Found migration defects can be a consequence of disturbed actin dynamics. We used a specific fluorescent dye to label F-actin filaments of both PD and control CD3/CD28-activated CD4^+^ T cells. Confocal imaging showed no difference in actin turnover between PD and the control group, suggesting other explanations for the observed migration defects (Supplementary Fig. 2).

### Alteration in mitochondrial functionality

**Fig. 3:**
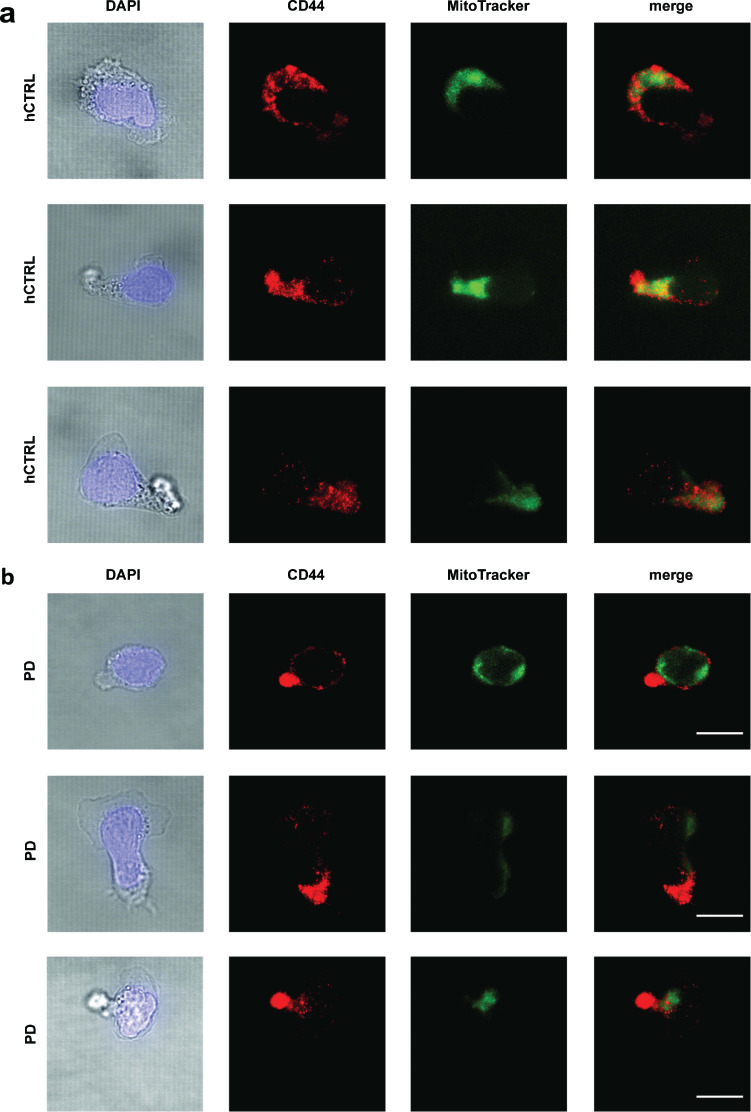
Impaired mitochondrial aligning with uropod in PD cells. Primary CD4^+^ T cells derived from three PD patients and three age/sex-matched healthy subjects were cultured for 7 days, transferred to human ICAM-1 coated µ-slides (10 μg/mL) and subsequently incubated for 45 min at 37 °C. Thereafter the cells were fixed, permeabilized and stained. **a** Representative brightfield and confocal images of DAPI (blue), MitoTracker (green), and CD44 (red) in single CD4^+^ T cells derived from healthy subjects and **b** PD patients. The images are obtained from three independent experiments. Scale bar, 10 μm.

Lymphocyte migration is shown to be regulated by mitochondria positioning at the posterior part of the cell, known as the trailing edge or uropod^17^. In order to test mitochondrial alignment with uropod in both PD and healthy control CD4^+^ T cells, we performed ICC using CD44 as a specific marker for uropod and MitoTracker green as a marker for mitochondria. As shown in Fig. 3, impaired alignment of mitochondria and uropod was observed in cells derived from PD patients.

**Fig. 4:**
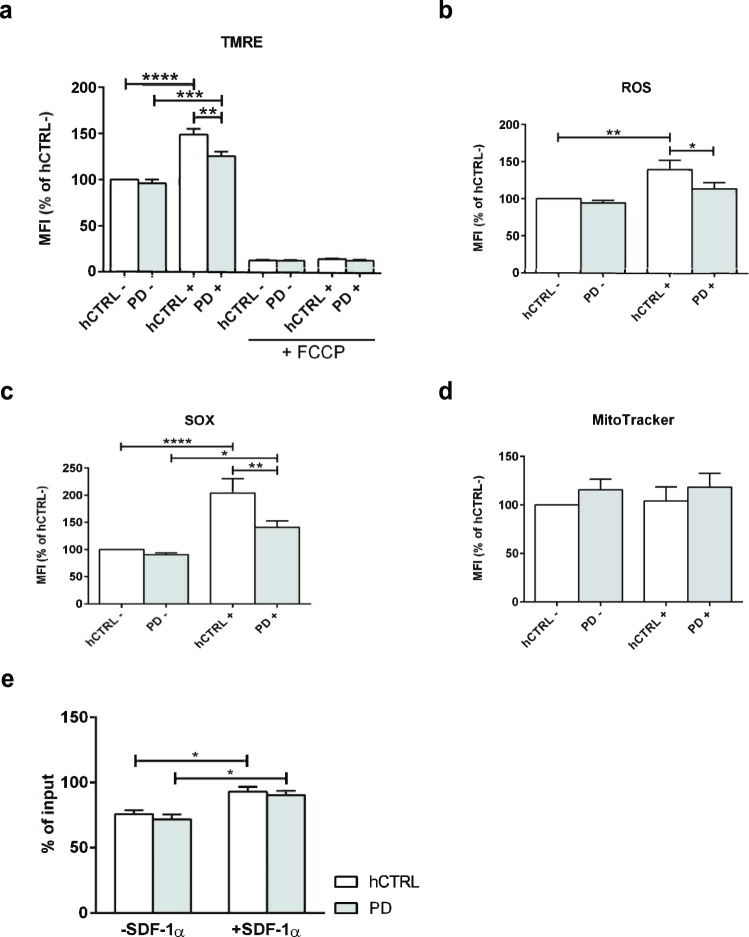
Altered mitochondrial activity in PD cells and Coenzyme Q10 rescue. Eleven PD (CD3/CD28 non-activated and activated, PD− and PD + respectively) and 11 age/sex-matched healthy controls (CD3/CD28 non-activated and activated, hCTRL− and hCTRL+ respectively) derived CD4^+^ T cells were cultured for 48 h, stained for viability and assayed accordingly. **a** Quantification of mitochondrial membrane potential (TMRE). The cells were stained with 50 nM TMRE for 20 min at 37 °C. The cells treated with 20 μM FCCP for 10 min at 37 °C were used as a control. TMRE signal was analysed by flow cytometry. **b** Quantification of DCFDA signal representing total cellular ROS production. The cells were stained with 20 μM DCFDA for 30 min at 37 °C and fluorescence was analysed by flow cytometry. **c** Quantification of mitochondrial superoxide (SOX). The cells were stained with 5 μM MitoSOX™ Red for 10 min at 37 °C and the signal was obtained by flow cytometry. **d** Quantification of MitoTracker. The cells were stained with 50 nM MitoTracker® Green FM for 30 min at 37 °C. The resulting fluorescence signal was analysed by flow cytometry. The data were obtained from 11 independent experiments. **a**–**d** Within each experiment, cumulative median fluorescence intensity data were expressed as a percentage of the hCTRL- group and shown as mean ± SEM. Statistical significance was assessed by using two-way ANOVA followed by Bonferroni’s multiple comparisons test. *p < 0.05, **p < 0.01, ***p < 0.001, ****p < 0.0001. **e** Transwell migration of Coenzyme Q10 treated primary CD4^+^ T cells derived from three PD patients and three age/sex-matched healthy controls with or without SDF-1α. The percentages of migrated cells to input cells for each group and condition are expressed as the mean percentage ± SEM. Statistical significance was assessed by using two-way ANOVA followed by Tukey’s multiple comparisons test. *p < 0.05.

To further assess whether spatial alteration of mitochondria impacts their functional properties, we used flow cytometry to analyze mitochondrial membrane potential and reactive oxygen species (ROS) production with and without CD3/CD28 activation in 11 PD and 11 controls. The mitochondrial membrane potential was assessed utilizing tetramethylrhodamine ethyl esters (TMRE), the total cellular ROS and mitochondrial superoxide (SOX), one of the most important ROS from which other ROS are derived^33^ and which has a significant role for stress signaling^34^. Our data revealed that all three parameters were significantly reduced in CD3/CD28-activated PD CD4^+^ T cells vs healthy control: TMRE (Interaction, *F*(1, 40) = 4.397 *p* = 0.0424; activation, *F* = 7098 *p* < 0.0001; PD, *F* = 8, 680 *p* = 0.0053); ROS (Interaction, *F*(1, 39) = 1.786 *p* = 0.1892; activation, *F* = 15,16 *p* = 0.0004; PD, *F* = 4457 *p* = 0.0412) and SOX (Interaction, *F*(1, 40) = 3456 *p* = 0.0704; activation, *F* = 2861 *p* < 0.0001; PD, *F* = 6319 *p* = 0.0161) (Fig. 4a–c).

To investigate the relationship between oxidative stress markers and clinical parameters, we correlated SOX, ROS, and TMRE levels in PD with disease stage, disease duration or levodopa medication, but found no significant correlations (Supplementary Fig. 1, spearman’s rho *p* > 0.05). We did, however, observe significant correlations between SOX, ROS, and TMRE in PD CD4^+^ T cells, which suggests these markers are interlinked. We finally analyzed the data separately for drug naïve and levodopa-treated PD patients, and noticed comparable changes of SOX and TMRE showing the same trend, while ROS was significantly reduced in activated CD4^+^ T cells derived from drug naïve PD patients (Interaction, *F*(1, 20) = 5107 *p* = 0.0351; activation, *F* = 7920 *p* = 0.0107; PD, *F* = 2737 *p* = 0.1137) (Supplementary Fig. 3).

We also quantified the total mitochondria content by analyzing MitoTracker with flow cytometry. There was no difference in mitochondrial quantification between PD-derived cells and control subjects with or without CD3/CD28 activation (Fig. 4d). Taken together, we can conclude that both improper mitochondria-positioning within the cells and their functional alteration may account for the migratory deficit observed in PD-derived CD4^+^ lymphocytes.

In order to restore observed defects, we have treated CD4^+^ T cells derived both from PD patients and age/sex-matched healthy controls with Coenzyme Q10 and performed the transwell assay with and without SDF-1α. Both groups increased the percentage of migrated cells to the lower chamber in the presence of chemokine, but there was no difference in percentages of migrated cells between the groups both with and without SDF-1α (Interaction, *F*(1, 8) = 0.0428 *p* = 0.8413; SDF-1α, *F* = 2702 *p* = 0.0008; PD, *F* = 0.9399 *p* = 0.3607). Coenzyme Q10 treatment of cells with described mitochondrial defect, provided a rescue (Fig. 4e).

Reduced ROS has been shown to lead to Th1-mediated immunity^35^ and a recent publication reported that PD-derived CD4^+^ T cells are Th1 biased^23^. In order to investigate the Th1 phenotype in our model system, we quantified the level of IFNγ in supernatants of CD4^+^ T cells derived from both PD and healthy individuals. Our data showed an increase in IFNγ level in cells originated from PD patients (hCTRL 4340 ± 4529 vs PD 6276 ± 6055 *p* = 0,0148) (Supplementary Fig. 4) corroborating a Th1 profile in PD.

Furthermore, we also compared CD3/CD28 activation level in both PD-derived cells and their healthy counterparts by performing surface staining for CD25 and CD69. Flow cytometry analysis showed no significant differences in CD69^+^CD25^−^, CD69^+^CD25^+^, and CD69^−^CD25^+^ subpopulations with or without activation, between PD and healthy controls, although there was a significant increase in percent of each subpopulation upon activation within each group (Supplementary Fig. 5).

### Cross-sectional study of p11 expression levels in CD4 + Th1/Th2/T17 cells

p11 is a neuronal protein known to associate with PD and L-dopa responses^24,27^ as well as depression and the action of antidepressant drugs^28,36^. Our previous publication showed that, apart from the brain, p11 is also expressed in peripheral blood cells, and its level has been increased in various populations of peripheral leukocytes derived from PD patients^24^. Protein p11 is an interacting partner of Annexin A2 and is found in the same complex^25^. Since Annexin A2 regulates actin bundling^26^, p11 may also have a role affecting the actin cytoskeleton. It has also been shown that the p11 gene and protein expression in human epithelial cells are induced by IFNγ^37^. In the present study, the intention was to characterize the expression level and distribution of p11 in different subsets of CD4^+^ T cells in both PD and control individuals.

**Fig. 5:**
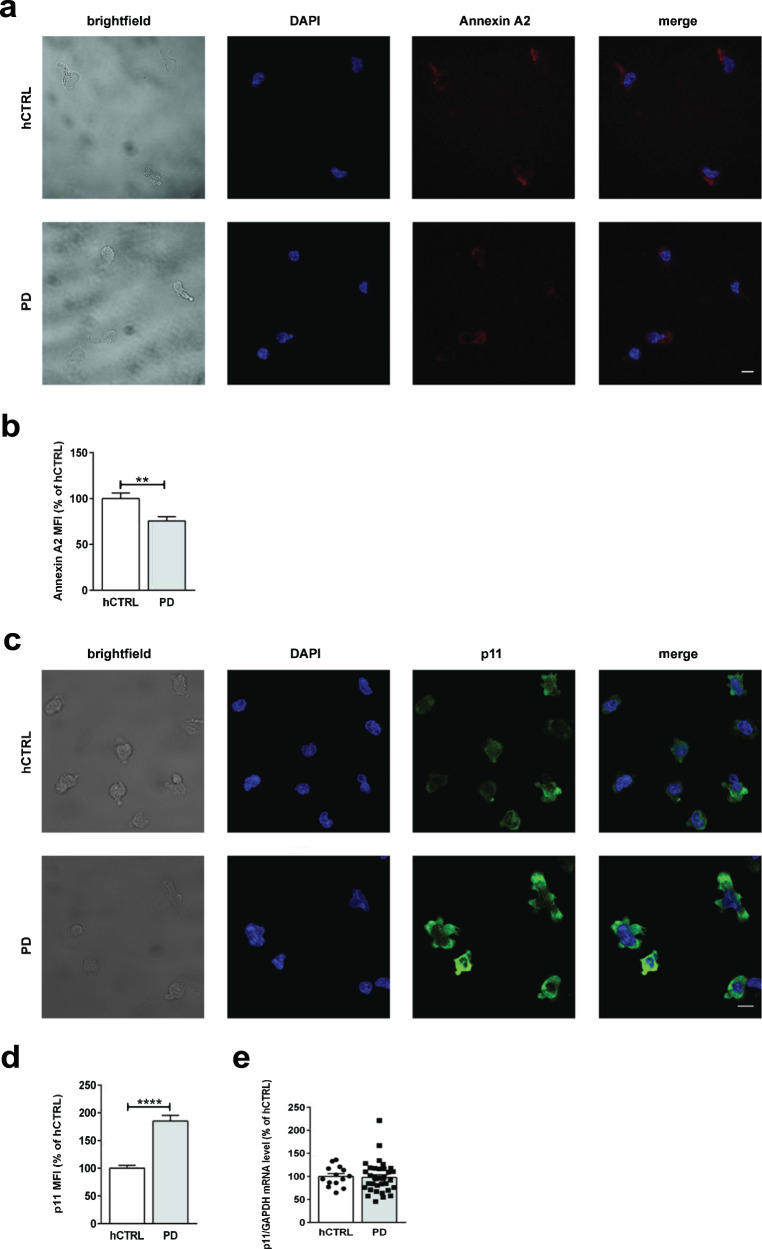
Decreased Annexin A2 and increased p11 protein expression levels in PD-derived CD4^+^ T cells. **a** Representative brightfield and confocal images of CD3/CD28-activated CD4^+^ T cells. Cells were fixed and stained for Annexin A2 (red) and DAPI (blue). Scale bar, 10 μm. **b** Quantification of Annexin A2 IF signal. Graphs represent mean ± SEM obtained from three independent experiments, containing three PD patients and three age/sex-matched healthy controls. In each experiment signal was normalized to the mean of the hCTRL cells. Statistical significance was assessed by Mann–Whitney test. **p < 0.01. **c** Representative brightfield and confocal images of CD3/CD28 activated CD4^+^ T cells. Cells were fixed and stained for p11 (green) and DAPI (blue). Scale bar, 10 μm. **d** Quantification of p11 IF signal. Graphs represent mean ± SEM obtained from ten independent experiments, containing ten PD patients and ten age/sex-matched healthy controls. In each experiment, signal was normalized to the mean of the hCTRL cells. Statistical significance was assessed by Mann–Whitney test. ****p < 0.0001. **e** qPCR analysis of p11 in PBMCs derived from 35 PD patients and 14 healthy control subjects. mRNA levels of p11 were normalized against GAPDH. Significance was assessed by Mann–Whitney test.

Purified CD4^+^ T cells were activated by priming with CD3/CD28 and subsequently stained with Annexin A2 antibody. There was 25% reduction in Annexin A2 protein level in activated PD cells as observed by confocal imaging (hCTRL 1000 ± 603 vs PD 7562 ± 441 *p* = 0.0014) (Fig. 5a, b). Purified CD4^+^ T cells, activated with CD3/CD28 were stained with p11 antibody. Confocal microscopy showed more than 80% increase in p11 expression level in activated PD-derived CD4^+^ T cells (hCTRL 1000 ± 523 vs PD 1854 ± 1008 *p* < 0.0001) (Fig. 5c, d) although there was no difference in p11 mRNA level between analyzed groups (Fig. 5e).

**Fig. 6:**
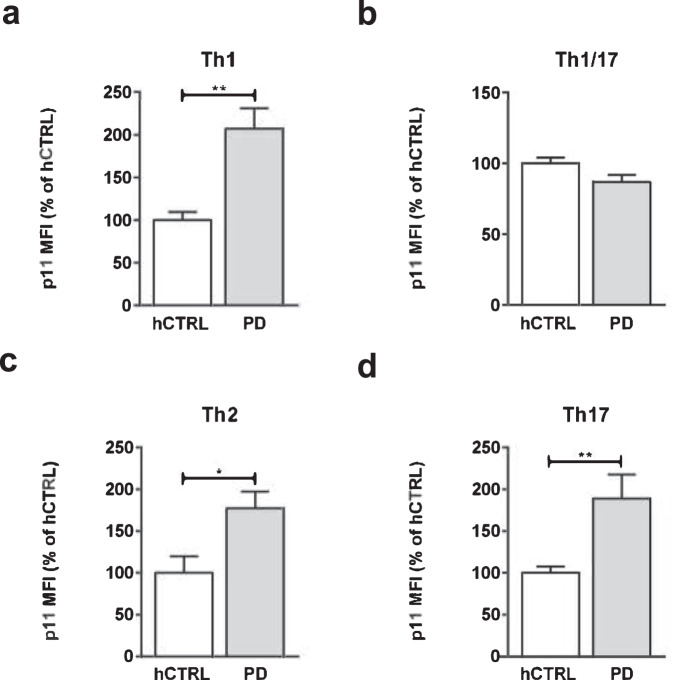
Specific p11 expression levels in PD-derived CD4^+^ Th1/Th2/T17 cells. PBMCs derived from 32 PD patients and 16 healthy control subjects were fixed, permeabilized and stained with monoclonal mouse anti-annexin II light chain (p11) antibody. Subsequently, the cells were stained for the following surface markers: CD3 FITC clone UCHT1, CD4 PE clone OKT4, CXCR3 BV510 clone 1C6, CCR3 APC-Cy7 Clone: 5E8, CCR4 PE-Cy7 Clone: 1G1, CCR6 PerCP Cy5.5® clone G034E3, analyzed by FACS and gated towards relevant Th subsets. Quantification of p11 expression level in **a** Th1, **b** Th1/17, **c** Th2, and **d** Th17 in PD-derived cells and healthy controls. The data are obtained from six independent experiments. Cumulative median FI data were normalized to the mean of the hCTRL group and expressed as mean ± SEM, within each experiment. Statistical significance was assessed by using Mann–Whitney test (Th1, Th1/17, and Th17) or Student’s *t*-test (Th2). *p < 0.05, **p < 0.01.

In order to determine the expression level of p11 protein in CD4^+^ T cell Th1, Th2 and Th17 cell populations, PBMCs derived from 32 PD and 16 control individuals (Suppl. Table 2) were stained and analyzed by flow cytometry (Supplementary Fig. 6). As shown in Fig. 6, our data revealed that p11 was significantly increased in Th1 (hCTRL 1000 ± 955 vs PD 2073 ± 2363 *p* = 0,0021), Th2 (hCTRL 1000 ± 1972 vs PD 17,740 ± 2005 *p* = 0.0155) and Th17 cells (hCTRL 1000 ± 758 vs PD 1888 ± 2909 *p* = 0,0040) derived from PD patients when compared to control cells, but this increase seems to be most prominent in the Th1 subset (Fig. 6a, c, d). However, there was no difference in p11 expression within the Th1/17 subset between PD and control groups (Fig. 6b).

## Discussion

Growing evidence suggests that adaptive immunity-related neuroinflammation contributes to PD, although the precise modes of action are yet to be unraveled^6^. It has been shown that human dopaminergic substantia nigra and stem cell-derived dopamine neurons express MHC-I, thereby becoming possible targets for T cell-mediated immunity in case of specific epitope display^38^. CD4^+^ T cells play a pivotal role in adaptive immunity^8,10^ therefore, any of their functional impairments may contribute to the inflammatory profile seen in PD. It has also been shown that PD patients have functional Th1 bias with specific cytokine profile^39^. Another study found increased frequency of IFNγ^+^ CD8^+^ T cells, IL-4^+^ Th2 cells and IL-17A^+^ Th17 cells in PD patients^40^. Detrimental effect of IL-17 resulting from neuronal death is suggested as one of mechanisms underlying PD pathogenesis characterized by increased cell counts of Th17 cells in the blood of patients and T lymphocytes in postmortem PD brains^41^. Nevertheless, to our knowledge, there are no data exploring the migration phenotype of CD4^+^ T cells, which may critically be involved in immune system dysfunction observed in PD patients. Proper migration of CD4^+^ T cells is important for competent immunological scanning of the environment and maintaining homeostasis. Murine studies demonstrated that impairment in migration phenotype introduces serious developmental defects^42^ that may lead to an immunocompromised profile.

In the present study, we showed that PD-derived cells display migratory deficits characterized by velocity, directionality and displacement reduction, suggesting improper functionality of T cell-mediated immunity. An earlier study has revealed that mitochondrial relocation at the uropod is critical for lymphocyte migration as their concentration at the uropod regulates the motor of migrating cells^14^. In our study, we report imaging data of impaired alignment of mitochondria with uropod in PD-derived cells, suggesting that improper mitochondria redistribution within these cells may represent the part of the underlying cellular mechanism of an observed migratory phenotype. This is in line with mitochondrial microtubule-dependent trafficking which has been implied in PD. Namely, disturbed mitochondrial function in PD does not sufficiently support the transport along microtubules, resulting both in impaired transport of autophagosomes to lysosomes for degradation and inefficient recruitment of mitochondria where needed in the cell^43–45^. We further proceeded with the functional investigation and found that PD-derived CD4^+^T cells show significant reduction of mitochondrial functional parameters such as ROS, SOX and mitochondrial membrane potential. These alterations may contribute to less competent adaptive immunity and increased infection incidence in PD patients. Indeed, it has been shown that, together with impaired swallowing and urine bladder emptying dysfunctions, PD patients have an increased risk of respiratory and urinary tract infections^46^.

Observed mitochondrial defects were restored by Coenzyme Q10 treatment resulting in normal migratory capacity of PD-derived CD4^+^ T cells. The migration level was equal to control regardless of chemokine conditions, further confirming that mitochondrial defect has an impact on the migration of PD cells.

In addition, the precise control of ROS production is crucial for regulating the appropriate signal transduction and T cell activation^17,47^. It is known that oxidative stress promotes the differentiation of human T cells toward Th2 phenotype^22^. Reduced homeostatic ROS production observed in our study drives Th1 bias in affected PD cells. By assessing IFNγ in supernatants of activated CD4^+^ T cells from PD patients and control subjects, we showed an increase in IFNγ production in cells originated from PD patients, which is in line with the previous publications^23^.

The PD and control subjects in each experiment were carefully age and sex-matched. Other covariates within the PD group, such as disease duration, disease stage, motor symptoms, and levodopa medication were explored, but no significant correlations were found between CD4^+^ T cell migration/mitochondrial parameters and clinical parameters. These findings suggest that migration and mitochondrial alterations we found are not affected by dopaminergic medication, nor change over the course of the disease. This is in accordance with other functional T cell studies that did not find any effect of dopaminergic medication on T cell function in PD^23^.

Besides observed mitochondrial defects, we investigated actin dynamics. Confocal imaging showed no difference in F-actin turnover between PD and control samples suggesting other actin regulation. Annexin A2 is a regulator of actin bundling coupling cytoskeleton with the plasma membrane and it is found either as a monomer or in a heterotetrameric complex with p11^26^. Also the role of p11 as a possible PD biomarker was suggested in our previous study that showed a differential expression pattern of p11 in various populations of peripheral leukocytes^24^. Here we investigated the expression profile of p11 in activated CD4^+^ T cells more deeply. Our data revealed an overall increase in p11 level in PD cells vs controls suggesting that either its synthesis or degradation may be altered. Since p11 mRNA expression was unchanged between PD and control cells, that may suggest that degradation mechanism is hampered in PD cells. Considering that mitochondrial dysfunction is shown to impair both proteasome assembly and activity^48^, the observed increase of p11 levels in PD might be a consequence of disturbed mitochondria. Moreover, our findings of elevated levels of both IFNγ and p11 in CD4^+^ T cells derived from PD patients are in line with previously published data showing that IFNγ increases p11 protein level in a time and dose-dependent manner^37^. Taken together, a simultaneous decrease in Annexin A2 and an increase in p11 protein levels can change complex stoichiometry, disturb actin regulation and contribute to the migration phenotype of PD-derived CD4^+^ T cells.

In order to analyse if p11 expression is more specifically distributed across various Th populations, we performed a cross-sectional study in different Th subsets. Previously we showed that there was no significant difference in the levels of p11 in T regulatory cells from PD and control subjects^24^. Hereby, we demonstrated that p11 expression level was elevated in the Th1, Th2, and Th17 subsets, while there was no difference within Th1/17 cell population when compared to control group. Upregulation of p11 in Th1 cells, Th1 bias together with increased IFNγ production but also specific increase level of p11 in Th2 and Th17 subsets suggests a potential of p11 to be used as a differential biomarker for PD. This work emphasizes that investigations in larger patient cohorts are needed to further elucidate the role of p11 in CD4^+^T cells and its alteration in PD.

One limitation of this study was the small cohort size in each of the experiments performed. Owing to limited sample availability, we were not able to perform all experiments on all subjects, hence we could not analyze the direct link between mitochondrial function, oxidative stress, p11, and migratory function in the same individuals. This also means the correlations between clinical parameters and functional experiments should be interpreted with caution. However, on the group level, the effect size of most experiments was large enough to make up for the limited sample size. In this study, we decided to focus on the role of CD4^+^ T cells in PD, however, the functional changes found could very well be extended to other immune cells and non-immune cells and should be explored in other follow-up studies.

Taken together, this work highlights functional changes in peripheral CD4^+^ T cells in PD. In particular, we show that compared to healthy subjects, CD4^+^ T cells derived from PD patients have altered migration potential along with differentially increased p11 levels across Th subsets, impaired mitochondrial positioning within the cell and reduced mitochondrial functionality. These results warrant replication in another larger PD cohort.

## Methods

### Ethics statement

The whole protocol used in this study was approved by the Regional Ethics Review Board of Stockholm (2014/1366-31). The study participants gave written informed consent in accordance with the Declaration of Helsinki.

### PBMC preparation and flow cytometry

PBMCs were isolated from the whole blood by using Lymphoprep™ density gradient medium (STEMCELL Technologies, #07851) as recommended by the vendor, frozen in medium containing 90% fetal bovine serum and 10% dimethyl sulfoxide and stored in liquid nitrogen before carrying out an appropriate assay. Processing of PBMCs and flow cytometry analysis of p11 have been essentially performed as previously published^24^. Frozen PBMC samples were quickly thawed, washed in PBS and total leukocytes were counted by digital hemocytometer, whereas cell viability was determined by the Trypan blue exclusion test (usually >90%) or stained for viability by using violet dead cell marker (Thermo Fisher Scientific, #L34963). Cells were washed again, fixed and permeabilized for 20 min by using Cytofix/Cytoperm™ solution (BD, #554722) and washed with Perm/Wash™ buffer (BD, #554723). Subsequently, the cells were stained with monoclonal mouse anti-annexin II light chain (p11) antibody (5 μg/mL, clone 148; BD Transduction Laboratories™, #610071) or isotype control mouse IgG1 antibody (5 μg/mL, clone MOPC-21; BioLegend, #400124) for 45 min at room temperature (RT). After the cells were washed with Perm/Wash™, a secondary APC- conjugated goat anti-mouse antibody (2 μg/mL; BioLegend, #405308) was applied for 30 min at RT, washed with Perm/Wash™ and blocked in buffer containing 1% mouse serum and 1% FBS in PBS. In order to identify CD4^+^ T helper cells and relevant subsets, the cells were stained with the following surface marker antibodies: CD3 FITC clone UCHT1, CD4 PE clone OKT4, CXCR3 BV510 clone 1C6, CCR3 APC-Cy7 Clone: 5E8, CCR4 PE-Cy7 Clone: 1G1, CCR6 PerCP Cy5.5® clone G034E3, followed by additional washing. The stained cells were analyzed using the multicolor flow cytometer FACS CantoII (BD). Quantification of p11 was based on the gating strategy shown in suppl. Fig. 6. Data sets were further analyzed with FlowJo 10.7.2 (Tree Star).

### Purification of CD4^+^ T Cells, activation, and immunofluorescence labeling

Peripheral blood mononuclear cells (PBMCs) were prepared as previously published^24^. CD4^+^ T Cell Isolation Kit (Miltenyi Biotec, #130-096-533) and LS Columns (Miltenyi Biotec, #130-042-401) were used for CD4^+^ T cells isolation according to manufacturer’s instructions and the culture purity was equal or above 95%. Isolated cells were resuspended in proliferation medium (RPMI 1640 with stable glutamine, Pen/Strep, heat-inactivated FCS, b-mercaptoethanol, non-essential amino acids, sodium pyruvate with freshly added interleukins IL-2 (Thermo Fisher Scientific, #PHC0026) and IL-7 (Thermo Fisher Scientific, #PHC0073). The cells with a density of 5 × 10^5^ cells/ml were seeded in 96-well plates which were pre-coated with 5 μg/mL anti-CD3 (clone OKT3), and 1 μg/mL anti-CD28 (clone CD28.2) antibodies to activate the cells. The cells were cultured for 2–7 days at 37 °C, 5% CO_2_ and assayed accordingly. Thereafter 7 days, cultured cells were transferred to µ-slides (Ibidi, #81501) previously coated with human ICAM-1 (10 μg/mL; R&D Systems, #720-IC-050), blocked with 2% BSA and washed with HBSS-based buffer (without Ca^2+^/Mg^2+^, supplemented with 0.2% BSA). Incubation was done for additional 45 min at 37 °C. After that the cells were fixed and permeabilized for 20 min by using Cytofix/Cytoperm solution (BD Biosciences) and washed 2x with Perm/Wash™. The cells were stained either with primary antibodies overnight at 4 °C (Annexin A2 rabbit monoclonal antibody, 1:10, Cell Signaling, #8235; p11 antibody, 1:50, BD, # 610071; rabbit monoclonal anti-CD44 antibody, 1:200, Abcam, # ab189524), with SPY555-actin at 1:1000 dilution for 1 h at RT or with MitoTracker® Green FM, 50 nM final conc. for 30 min at 37 °C. In experiments where MitoTracker® Green FM was combined with primary antibody, the staining with MitoTracker® Green FM was performed first. After the cells were washed 2x with Perm/Wash™, secondary antibodies goat anti-mouse Alexa Fluor^TM^ 488 (1:500, Thermo Fisher Scientific, #A11029) or donkey anti-rabbit Alexa Fluor^TM^ 568 (1:500, Invitrogen, #A10042) were applied for 1 h at RT. The cells were washed 2x with Perm/Wash™ and stained with DAPI (300 nM, Sigma-Aldrich).

### Confocal microscopy, image analysis, and live imaging

Acquisition of immunofluorescent images was done on ZEISS LSM 880 Airyscan confocal laser scanning microscope equipped with ZEN 2.1 software, using Plan- Apochromat 63x/1.4 Oil DIC M27 63x oil objective. The quantification analysis was done by using ImageJ. Image thresholding was used to define the outline of the cell and immunofluorescence signal intensity was obtained by using sum projection of Z stack images after background has been subtracted. For each experiment, PD group data were normalized to the mean fluorescence intensity of control group and expressed as % of the mean ± SEM. The data of p11 expression in CD4^+^ T cells were pooled from ten independent experiments where ten subjects in total per each group were analyzed.

In order to perform live imaging, 7 days cultured cells from one well from 96-well plate were harvested, spun down by centrifuging and resuspended in migration buffer containing HBSS with Ca^2+^/Mg^2+^ (1 mM each), 10 mM HEPES and 0.2% BSA. 50 μl of cells were transferred per well of coated µ-slides prepared as described above, incubated at 37 °C, 5% CO_2_ for 45 min. Acquisition of movies was done on the same microscope by using a 10x objective for brightfield cell tracking. Manual cell tracking was carried out by applying a manual tracking plugin for ImageJ at 10x for 20 min per each recording. Generated results were analyzed with Ibidi chemotaxis and migration tool version 2.0.

### Transwell migration

In order to assay CD4^+^ T cell migration, 1 × 10^5^ cells in migration buffer containing RPMI 1640 with stable glutamine, with antibiotics, 10 mM HEPES and 0.25 % fatty acid-free BSA (Sigma-Aldrich, #A7030), were serum starved for 30 min at 37 °C. Transmigration to SDF-1α (R&D Systems, #350-NS-010) was assayed by using Costar 5 μm pore size transwell plates. Transwell membranes were coated with human ICAM-1 (10 μg/mL), blocked with 2% BSA and washed. The cells were left to migrate for 1 h at 37 °C to the lower chamber and were subsequently counted by FACS. Input controls were used to calculate the migration rate. The data were expressed as percentage of the input controls.

### ELISA

IFNγ ELISA was performed by using a Human IFNγ ELISA Kit (Thermo Fisher Scientific, #EHIFNG). Supernatants of cultured PD and control CD4 + T cells were harvested after 48 h of CD3/CD28 activation and frozen at −80 °C until analysis. Prior to assay, all samples were thawed and processed according to the manufacturer’s recommendations. In total, 19 subjects per each group were analyzed.

### Functional assessment of mitochondria by flow cytometry

CD4^+^ T cells were cultured for 48 h. The cells (1 × 10^5^) were stained for viability either by using a UV dead cell marker (Thermo Fisher Scientific, #L34961) followed by activation assay or Near-IR dead cell marker (Thermo Fisher Scientific, #L10119) followed by mitochondria functional assays. Stained cells were analyzed by using the multicolor flow cytometer Gallios (Beckman Coulter). Data sets were further analyzed with Flow Jo10.7.2 (Tree Star).

#### Activation Control

After viability staining, cells were washed, stained with fluorophore-conjugated antibodies against the following surface markers: CD25 (clone 2A3, PE-conjugated, BD, #341011) and CD69 (clone FN50, APC-conjugated, Biolegend, #310910) and incubated at +4 °C for 30 min. After final washing, stained cells were analyzed by flow cytometry.

#### Mitochondrial membrane potential

TMRE-Mitochondrial Membrane Potential Assay Kit (Abcam, #ab113852) was used for the quantification of mitochondrial membrane potential changes. TMRE (tetramethylrhodamine, ethyl ester) is a positively charged dye which accumulates in active mitochondria because of their relative negative charge. This assay includes control treatment with FCCP [carbonyl cyanide 4-(trifluoromethoxy) phenylhydrazone], which severely disturbs mitochondrial membrane potential and hence blocks TMRE staining. Control cells were treated with 20 μM FCCP for 10 min at 37 °C. All cells were then stained with 50 nM TMRE for 20 min at 37 °C without any further washing. The fluorescence of the TMRE signal was analysed in the PE channel by flow cytometry.

#### Cellular reactive oxygen species detection

DCFDA Cellular ROS Detection Assay Kit (Abcam, #ab113851) was used for ROS detection. Assay employs DCFDA (2′,7′– dichlorofluorescein diacetate) which is a fluorogenic dye. This dye is deacetylated by cellular esterases and oxidized by ROS into 2′,7′-dichlorofluorescein (DCF), a highly fluorescent compound. This process correlates with ROS activity. The cells were stained in a culture medium with 20 μM DCFDA for 30 min at 37 °C and instantly placed on ice after the staining without any further washing. Fluorescence was analysed in the FITC channel by flow cytometry.

#### MitoSOX-assay

A mitochondrial superoxide indicator for live-cell imaging, MitoSOX™ Red (Invitrogen, #M36008), was used for the detection of superoxide according to the manufacturer’s instructions. Briefly, the MitoSOX™ Red compound is specifically oxidized only by mitochondrial superoxide but not by other ROS, producing red fluorescence. The cells were stained in a culture medium with 5 μM MitoSOX™ Red compound for 10 min at 37 °C. After washing three times with a warm medium, fluorescence was analysed in the PE channel by flow cytometry.

#### MitoTracker

MitoTracker® Green FM (Invitrogen™, #M7514) was used for quantification of mitochondria. After washing in PBS, cells were stained with 50 nM MitoTracker® Green FM reagent for 30 min at 37 °C, washed and resuspended in PBS. The resulting fluorescence was analysed in the FITC channel by flow cytometry.

#### Coenzyme Q10 treatment

Isolated CD4^+^ T cells were cultured under CD3/CD28 activating conditions and 24 h prior the transwell assay, the cells were treated with Coenzyme Q10 (Tocris, #3003) at final concentration of 5 µM.

### Total RNA extraction and quantitative real-time polymerase chain reaction (qRT-PCR) in PBMCs

Total RNA was extracted from PBMCs (106–107 cells) using the RNeasy Plus Mini Kit (#74134, Qiagen). For PBMC samples with a cell number less than 106, RNeasy Micro Kit (#74004, Qiagen) was used. All steps were performed according to the manufacturer’s protocol. The concentration and purity of acquired RNA samples were determined by a Nanodrop (Marshall Scientific). All RNA samples were kept at −80 °C till use.

QuantiTect Reverse Transcription Kit (#205311, Qiagen) was applied to synthesize cDNA following the instructions. Transcription levels of p11 were measured by qRT- PCR. In short, reactions containing HOT FIREPol EvaGreen Supermix (08-36-00008, Solis Biodyne) were prepared in triplicate for each sample and run in a CFX96 Real-Time System (Bio-Rad). The reactions started with an initial activation at 95 °C for 12 min, followed by 40 cycles of denaturation (95 °C, 15 s), annealing (60 °C, 30 s), and elongation (72 °C, 30 s). Comparative quantification algorithm ΔΔCt was used to determine expression levels, in which GAPDH served as a reference gene for normalization. Primers were p11 (Forward: CCAAGGCTTCAACGGACCAC; Reverse: GCCAGAGGGTCTTTTTGATTTTCCA) and GAPDH (Forward: GACAGTCAGCCGCATCTTCT; Reverse: TTAAAAGCAGCCCTGGTGAC).

### Statistical analysis

GraphPad Prism 6 and RStudio (R version 4.0.2, packages corrplot and ggplot) were used to perform statistical analysis. All data in this study were presented as means ± standard error of means if not specified differently. In order to determine data distribution, D’Agostino & Pearson omnibus normality test was used. The data with normal distribution were assessed by using two-way ANOVA or parametric unpaired *t*-test while the data without normal distribution required using non-parametric Mann–Whitney test. The statistical significance level was set for *p* < 0.05.

## Supplementary information

Supplementary Material

## Data Availability

The data were available upon request, but the ethics protocol does not allow open sharing because of the sensitive nature of the patient’s clinical information, and Data Use Agreement will be needed.
