# Supplementary material for: Impaired migratory phenotype of CD4+ T cells in Parkinson’s disease
Source: NPJ Parkinsons Dis. 2022 Dec 10;8:171. doi: 10.1038/s41531-022-00438-0 (PMC9741605; doi:10.1038/s41531-022-00438-0)

Supplementary Table 1. Demographical and clinical characteristics of all participating subjects per each figure without p11 cross-sectional study (see Suppl. Table 2). PD, Parkinson's disease; F, female; M, male; n/a, not applicable; H&Y, Hoehn and Yahrscale; MADRS, Montgomery-Åsberg Depression Rating Scale; PBMCs,peripheral blood mononuclear cells. Measures are presented as mean ± standarddeviation, median (range) or median (interquartile range [IQR]).

PBMCs					
	Group	Gender (M/F) (%)	Age mean +/- SD	Disease duration (years) median (range)	H&Y score median (range)
Figure 1	Control (n = 11)	54/48	65,4±7,6	n/a	n/a
	PD (n = 11)	54/48	71,9±6,7	8,7 (1,7-21,1)	2 (1,5-5)
Figure 2	Control (n = 9)	56/44	67,0±8,5	n/a	n/a
	PD (n = 9)	56/44	65,4±8,5	5,4 (0,0-18,6)	2,8 (1-5)
Figure 3	Control (n = 3)	33/67	58,7±7,2	n/a	n/a
	PD (n = 3)	33/67	65,3±9,1	3,2 (1,8-10,1)	2,8 (1,5-4,0)
Figure 4 and Suppl. Fig. 5	Control (n = 11)	36/64	65,6±10,1	n/a	n/a
	PD (n = 11)	36/64	71,8±9,2	7,7 (1,8-21,1)	2,0 (1,5-3,0)
Figure 4E and Suppl. Fig. 2	Control (n = 3)	33/67	66,7±12,1	n/a	n/a
	PD (n = 3)	33/67	75,1±3,6	11,1 (1,1-21,1)	2 (2-3)
Figure	Control (n = 3)	33/67	58,3±10,4	n/a	n/a

5B					
	PD (n = 3)	33/67	59,7±5,0	3,2 (0,0-12,2)	2,5 (1,5-3,0)
Suppl. Fig. 3	Control (n = 6)	50/50	66,3±10,8	n/a	n/a
	PD (n = 6)	50/50	75,7±8,6	0,7 (0,3-1,2)	3,2 (1,5-4)
Suppl. Fig. 4	Control (n = 19)	47/53	64,4±9,1	n/a	n/a
	PD (n = 19)	47/53	68,2±9,8	7,6 (1,0-21,1)	2,0 (1,0-4,0)

PBMCs						
Figure 5D	Group	Gender (M/F) (%)	Age mean +/- SD	Disease duration (years) median (range)	H&Y score median (range)	MADRS median (IQR)
	Control (n = 10)	60/40	66,1±9,0	n/a	n/a	n/a
	PD (n = 10)	70/30	66,9±8,0	5,4 (0,0-18,6)	2,8 (1,0-5,0)	12,0 (8,2-17,2)

Supplementary Table 2. Demographical and clinical characteristics of all subjects who participated in cross-sectional study of p11 expression in CD4+ Th1/Th2/T17. PD, Parkinson's disease; F, female; M, male; n/a, not applicable; H&Y, Hoehn and Yahrscale; MADRS, Montgomery-Åsberg Depression Rating Scale; PBMCs,peripheral blood mononuclear cells. Measures are presented as mean ± standarddeviation, median (range) or median (interquartile range [IQR]).

PBMCs						
Figure 6	Group	Gender (M/F) (%)	Age mean +/- SD	Disease duration (years) median (range)	H&Y score median (range)	MADRS median (IQR)
	Control (n = 16)	62/38	63,9 +/- 11,2	n/a	n/a	n/a
	PD (n = 32)	69/31	64,8 +/- 9,9	4,8 (0 – 21,1)	2 (1 - 5)	8 (4-12,5)

Supplementary figure legends

Supplementary Figure 1: **Correlation analysis of clinical parameters and migration parameters/oxidative stress markers in PD derived CD4$^+$ T cells**

A) Correlation plot of clinical scales and migration parameters and p11 levels in 9 PD patients' CD4$^+$ T cells. Scales assessed were Hoehn & Yahr and Unified Parkinson's Disease Rating Scale (UPDRS) III for disease stage and motor symptoms, Levodopa Equivalent Daily Dose (LEDD) for medication, Montreal Cognitive Assessment (MoCA) for cognition, and Montgomery Åsberg Depression Rating Scale (MADRS) for depression. Migration parameters include velocity and directionality of CD4$^+$ T cells. The correlations were made using Spearman's rho, p values indicated with asterisks: *p<0.05, **p<0.01, ***p<0.001, ****p<0.0001. B) Correlation plot of clinical parameters and oxidative stress markers in 11 PD patients' CD4$^+$ T-cells. Scales assessed were Hoehn & Yahr and Unified Parkinson's Disease Rating Scale (UPDRS) III for disease stage and motor symptoms, Levodopa Equivalent Daily Dose (LEDD) for medication, and Montreal Cognitive Assessment (MoCA) for cognition. Oxidative stress markers were measured before and after CD4$^+$ T cell activation, and included reactive oxygen species (ROS), mitochondrial superoxide (SOX), and tetramethylrhodamine ethyl esters (TMRE). The correlations were made using Spearman's rho, p values indicated with asterisks: *p<0.05, **p<0.01, ***p<0.001, ****p<0.0001.

Supplementary Figure 2: **F-actin dynamics is in healthy range in PD derived CD4$^+$ T cells** A) Representative brightfield and confocal images of CD3/CD28 activated CD4$^+$ T cells derived from 3 PD patients and 3 age/gender matched healthy controls. Cells were fixed and stained for SPY555-actin (red) and DAPI (blue). Scale bar, 10 µm. B) Quantification of SPY555-actin IF signal. Graphs represent mean ± SEM obtained from 3 independent experiments. In each experiment signal was normalized to the mean of the hCTRL cells. Statistical significance was assessed by Mann–Whitney test.

Supplementary Figure 3: **Mitochondrial activity in CD4$^+$ T cells derived from drug naïve PD patients**

6 drug naïve PD patients (CD3/CD28 non-activated and activated, PD- and PD+ respectively) and 6 age/gender matched healthy control (CD3/CD28 non-activated and activated, hCTRL- and hCTRL+ respectively) derived $CD4^+$ T cells were cultured for 48h, stained for viability and assayed accordingly. A) Quantification of mitochondrial membrane potential (TMRE). The cells were stained with 50 nM TMRE for 20 min at 37°C. The cells treated with 20 μM FCCP for 10 min at 37°C were used as a control. TMRE signal was analysed by flow cytometry. B) Quantification of DCFDA signal representing totalcellular ROS production. The cells were stained with 20 μM DCFDA for 30 min at 37°C and fluorescence was analysed by flow cytometry. C) Quantification of mitochondrial superoxide (SOX). The cells were stained with 5 μM MitoSOX™ Red for 10 min at 37°Cand the signal was obtained by flow cytometry. A)-C) Within each experiment cumulative median fluorescence intensity data were expressed as percentage of the hCTRL- group and shown as mean ± SEM. Statistical significance was assessed by using two-way ANOVAfollowed by Bonferroni's multiple comparisons test. *$p<0.05$, **$p<0.01$.

Supplementary Figure 4: **Increased IFNγ production by PD derived $CD4^+$ T cells** IFNγ was measured in supernatants of PD and hCTRL derived $CD4^+$ T cells after 48h of CD3/CD28 activation. Data were presented as means ± SEM of 19 subjects per each group. PD patients and hCTRLs were age/gender matched. Statistics was done by using Student's t-test. *$p<0.05$.

Supplementary Figure 5: **Activation markers CD25 and CD69 upon CD3/CD28 activation in PD and healthy subjects derived $CD4^+$ T cells** PD (CD3/CD28 non-activated and activated, PD- and PD+ respectively) and healthy control (CD3/CD28 non-activated and activated, hCTRL- and hCTRL+ respectively) derived $CD4^+$ T cells were cultured for 48h, stained for viability and assayed accordingly. Surface expression of early activation markers CD25 (IL2Rα) and CD69 was measured by flow cytometry after 48h of CD3/CD28 activation. A) Representative FACS plots of PD and hCTRL cells with or without activation. The numbers in Q1-Q4 quadrants represent the percent of parent population. Surface expression of the early activation markers CD25

(IL2Rα) and CD69: B) Q1 ($CD69^+$ $CD25^-$), C) Q2 ($CD69^+$ $CD25^+$) and D) Q3 ($CD69^-$ $CD25^+$). The data are obtained from 11 PD patients and 11 age/gender matched hCTRLs. Cumulative percent of parent population data represented as mean ± SEM. Statistical significance was assessed by using two-way ANOVA followed by Tukey's multiple comparisons test. *p<0.05, ***p<0.001, ****p<0.0001.

Supplementary Figure 6: **Flow cytometry analysis of p11 expression level in $CD4^+$ Th1/Th2/T17 cells** Representative flow cytometry gating strategy plots of PBMCs stained with a multicolor antibody panel for the identification of Th1/Th2/Th17 subsets. The gating order is denoted with black arrows and comprises subsequent separation of lymphocytes, singlets, $CD4^+$ T cells with eventually gated Th1, Th1/17, Th2 and Th17 cells. Two overlaid histograms on the left, show intracellular staining of p11 in PD and healthy control cells with isotype control, denoted with green, red and light blue color respectively, within Th1 and Th2 subsets. One overlaid histogram on the far left, shows Th1 subset (grey) and Th2 subset (blue) analyzed for specific Th2 surface marker CCR3.

Supplementary Figure 1

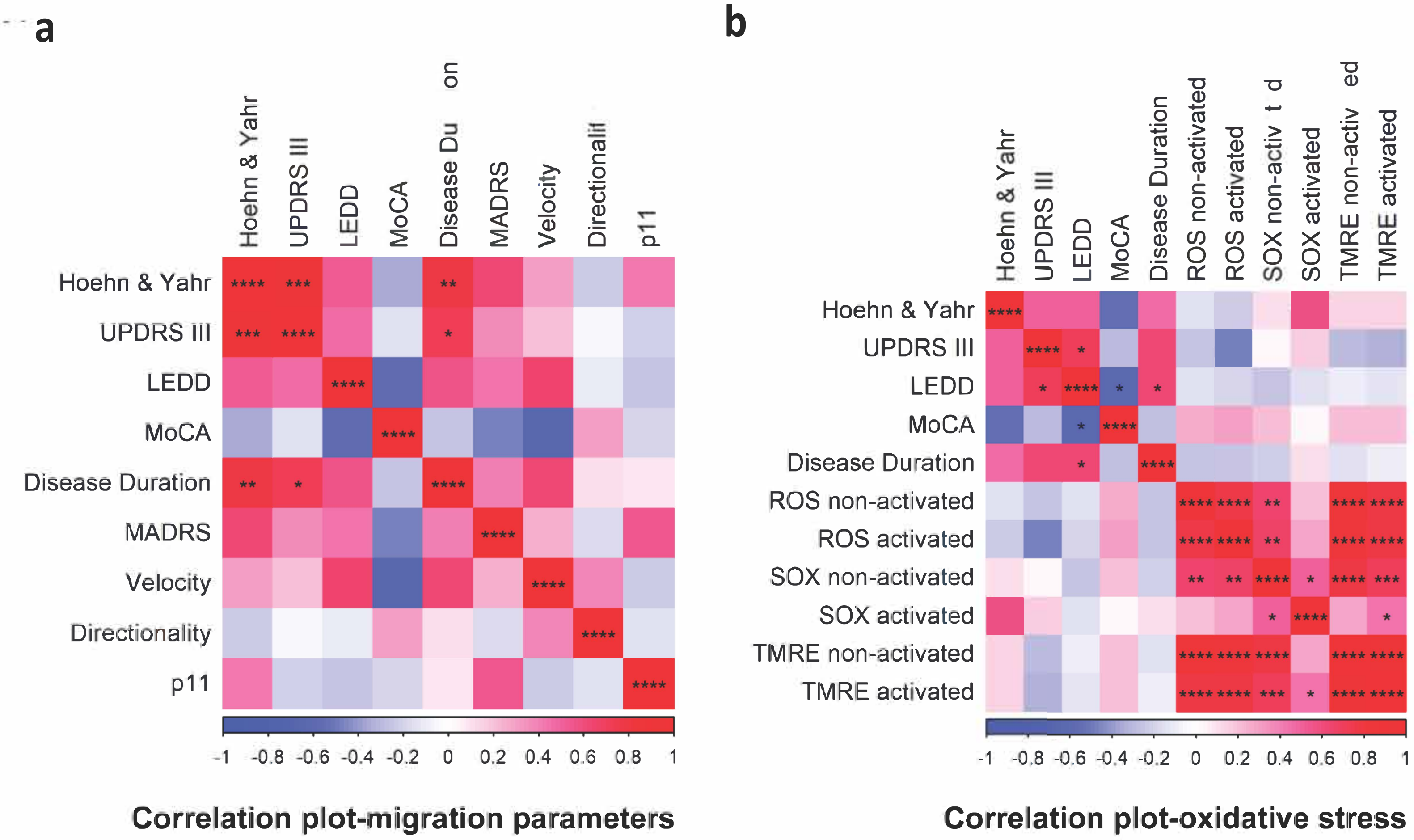

Supplementary Figure 2

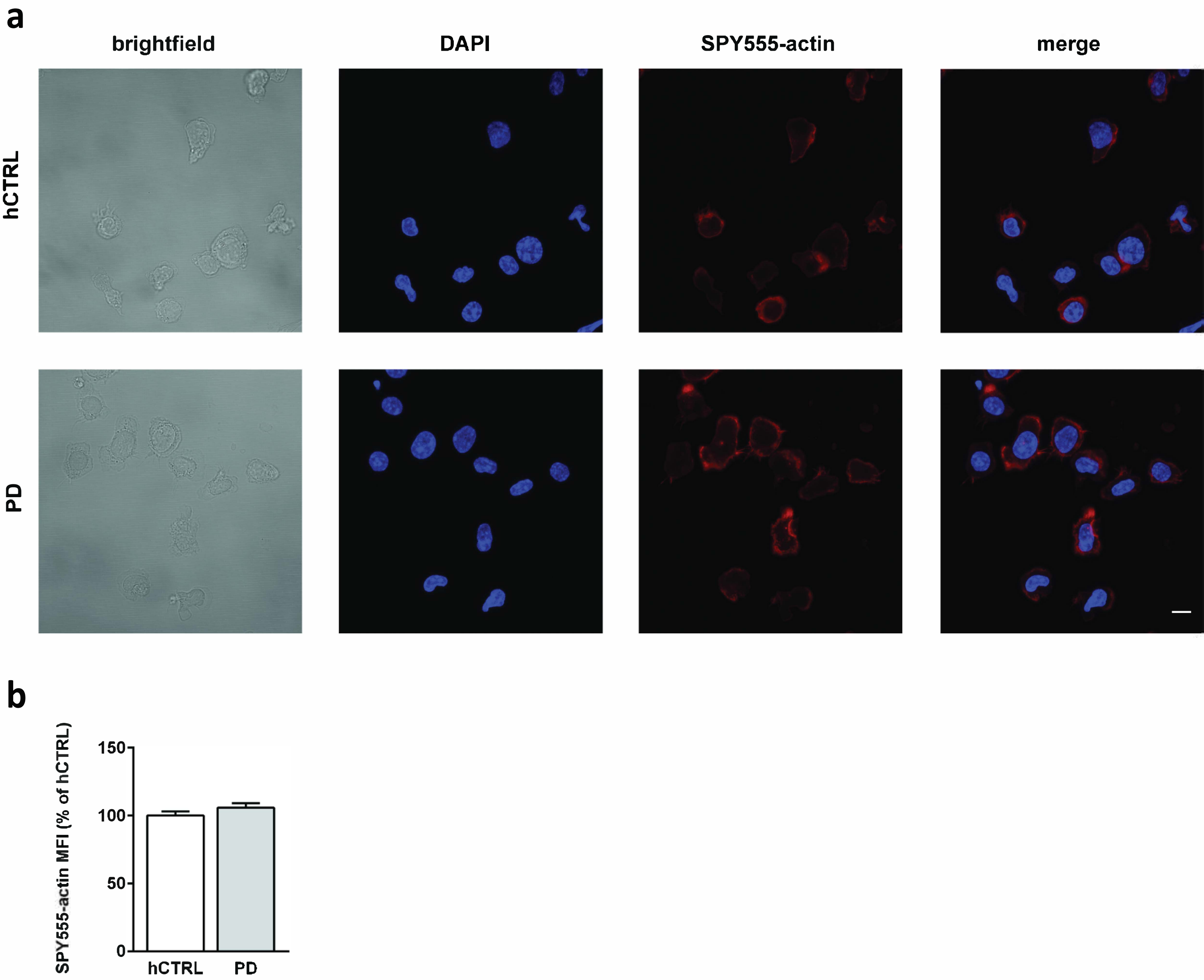

Supplementary Figure 3

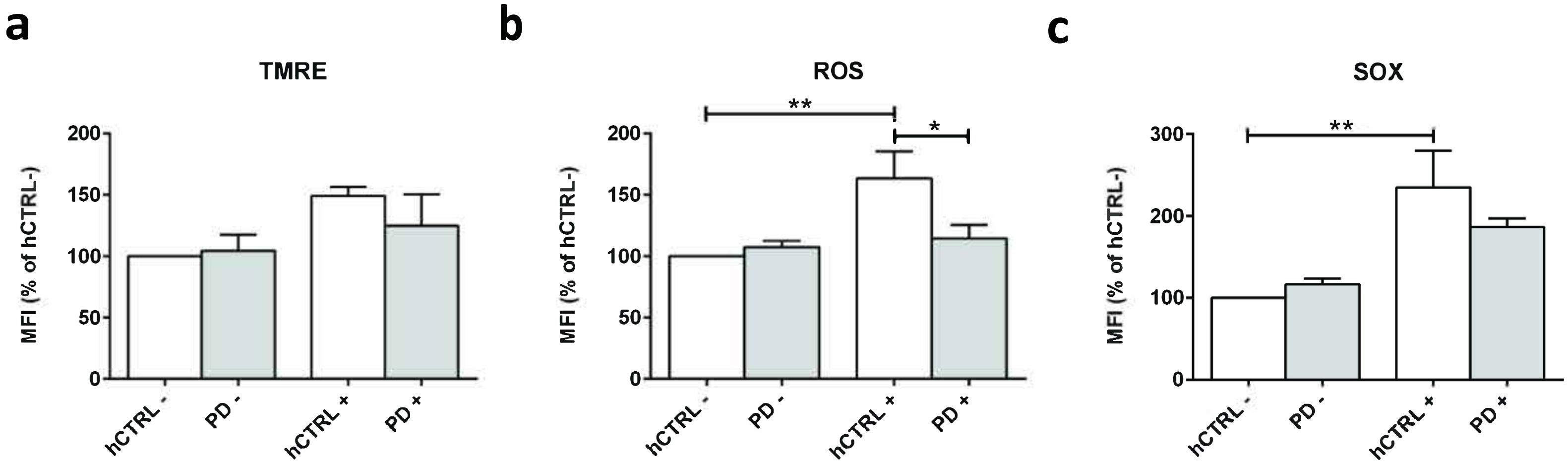

Supplementary Figure 4

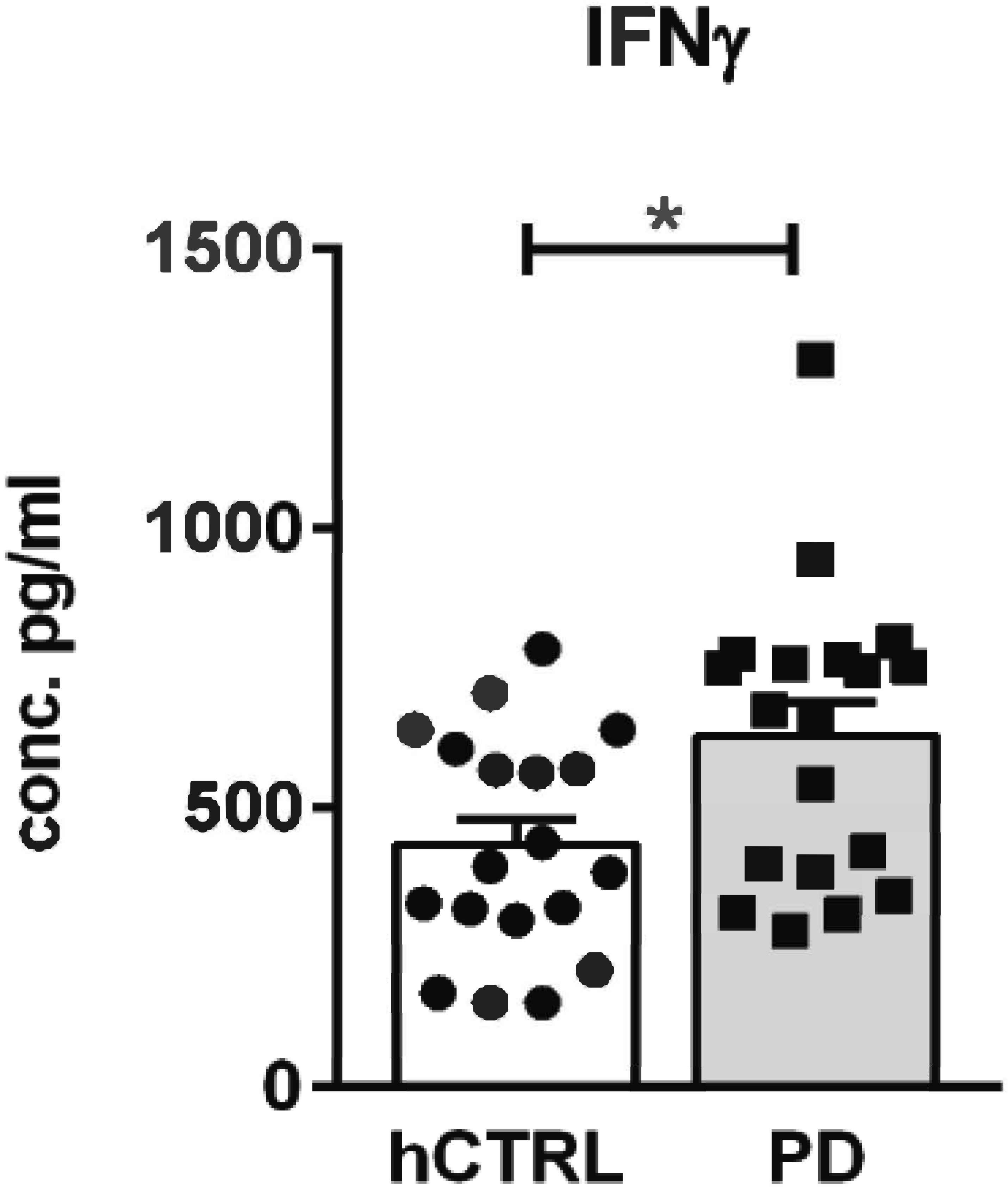

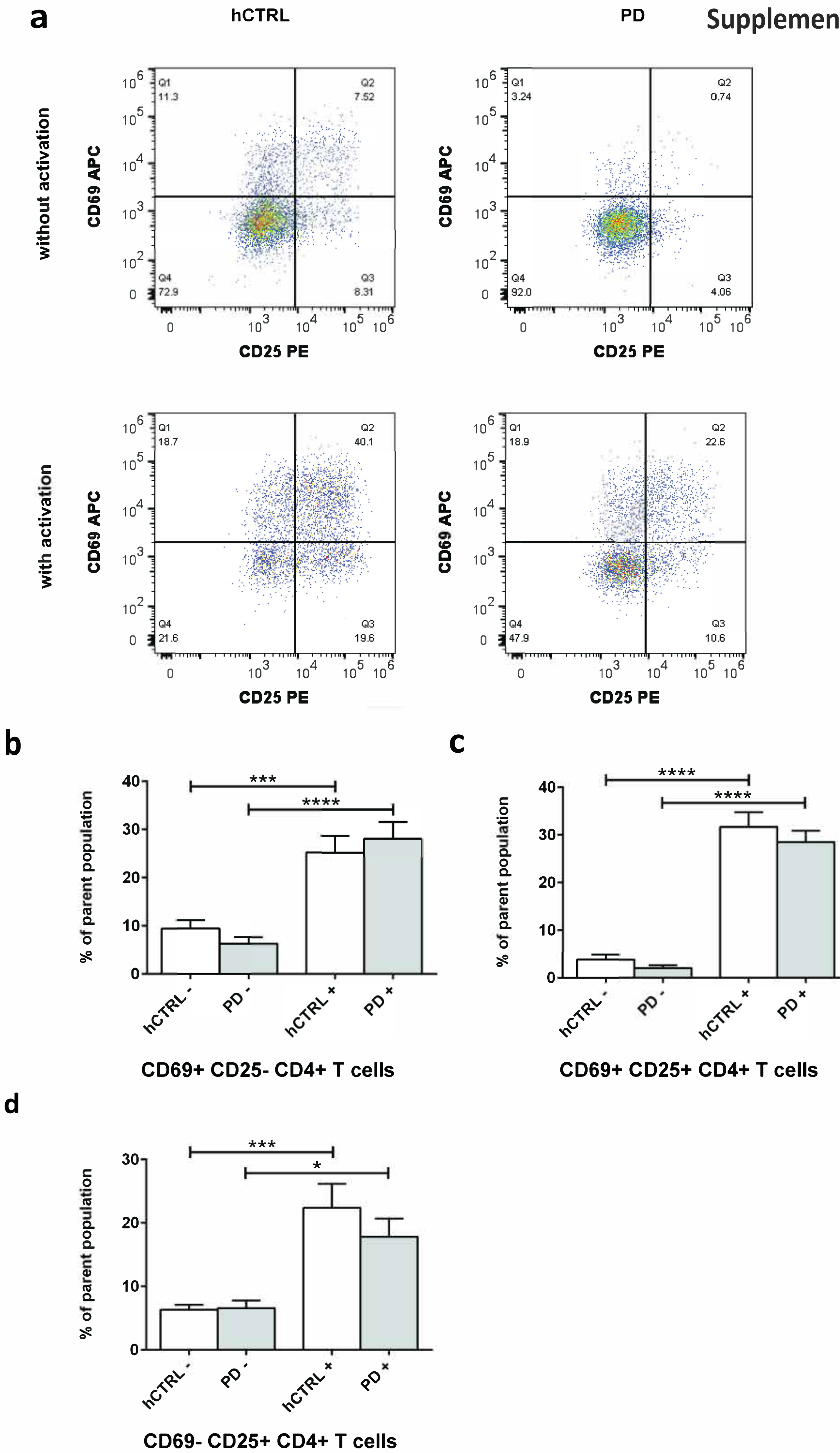
a
hCTRL
PD
without activation
with activation
CD69 APC
CD25 PE
Q1
11.3
Q2
7.52
Q4
72.9
Q3
8.31
Q1
3.24
Q2
0.74
Q4
92.0
Q3
4.06
Q1
18.7
Q2
40.1
Q4
21.6
Q3
19.6
Q1
18.9
Q2
22.6
Q4
47.9
Q3
10.6
b
% of parent population
***
****
hCTRL -
PD -
hCTRL +
PD +
CD69+ CD25- CD4+ T cells
c
****
****
CD69+ CD25+ CD4+ T cells
d
***
*
CD69- CD25+ CD4+ T cells

Supplementary Figure 6

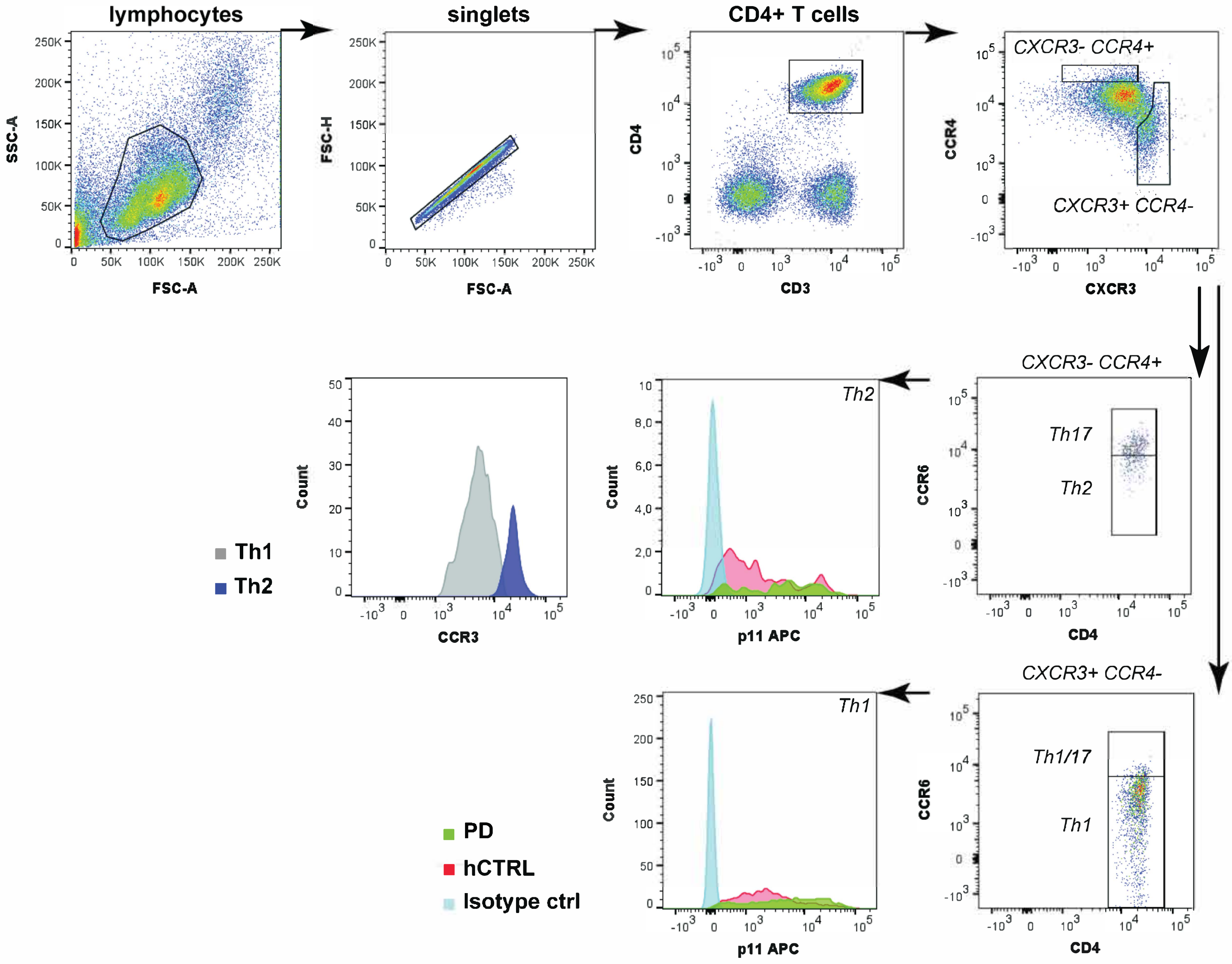

Supplement: Supplementary file 1 — Supplementary Material [file 41531_2022_438_MOESM1_ESM.pdf]
